# Diffractive wavefront correction for Fe *L*-edge spectroscopy on the meV scale

**DOI:** 10.1107/S1600577526005254

**Published:** 2026-06-16

**Authors:** Christoph Braig, Andrey Sokolov, Frank Siewert, Christian Seifert

**Affiliations:** a Institute for Applied Photonics eV, Rudower Chaussee 29/31, 12489Berlin, Germany; b Helmholtz-Zentrum Berlin für Materialien und Energie (HZB) Albert-Einstein-Str. 15 12489Berlin Germany; IOM-CNR and Elettra-Sincrotrone, Italy

**Keywords:** X-ray spectroscopy, diffractive optics, reflection zone plates, wavefront correction

## Abstract

We propose a wavelength-dispersive instrument for high-resolution soft X-ray spectroscopy at large-scale facilities like synchrotron radiation sources or free-electron lasers. Demonstrated by simulations at the Fe *L*-edge in the range from 700 eV to 730 eV, an energy resolution of (12–15) meV is enabled by a wavefront-corrected reflection zone plate (RZP) with an aperture of 40 mm × 190 mm on a spherical Si substrate (radius 69 m). Its tangential slope error and deformation are measured on-axis along the RZP to ±0.1 arcsec (r.m.s.) and ±6.1 nm (r.m.s.), respectively, and simulated at off-axis positions of the substrate with a similar amplitude. The surface waviness is compensated around the design energy of 715 eV by a two-dimensional, adapted grating groove distribution. As a benefit, the diffracted beam is collimated to a one-dimensional focal line whose length of 40 mm nearly equals the sagittal detector size (50 mm): no photons in the +1st diffraction order with an efficiency of 6.4% are lost, and the acceptance solid angle of 16 mrad × 3.3 mrad provides a high transmission of the signal from the source.

## Abstract 2

We propose an aberration-corrected, wavelength-dispersive instrument for soft X-ray spectroscopy at an energy-resolving power of (5–6) × 10^4^, designed for, for example, resonant inelastic X-ray scattering at synchrotron beamlines or X-ray free-electron lasers.

## 1. Introduction

**Figure 1:**
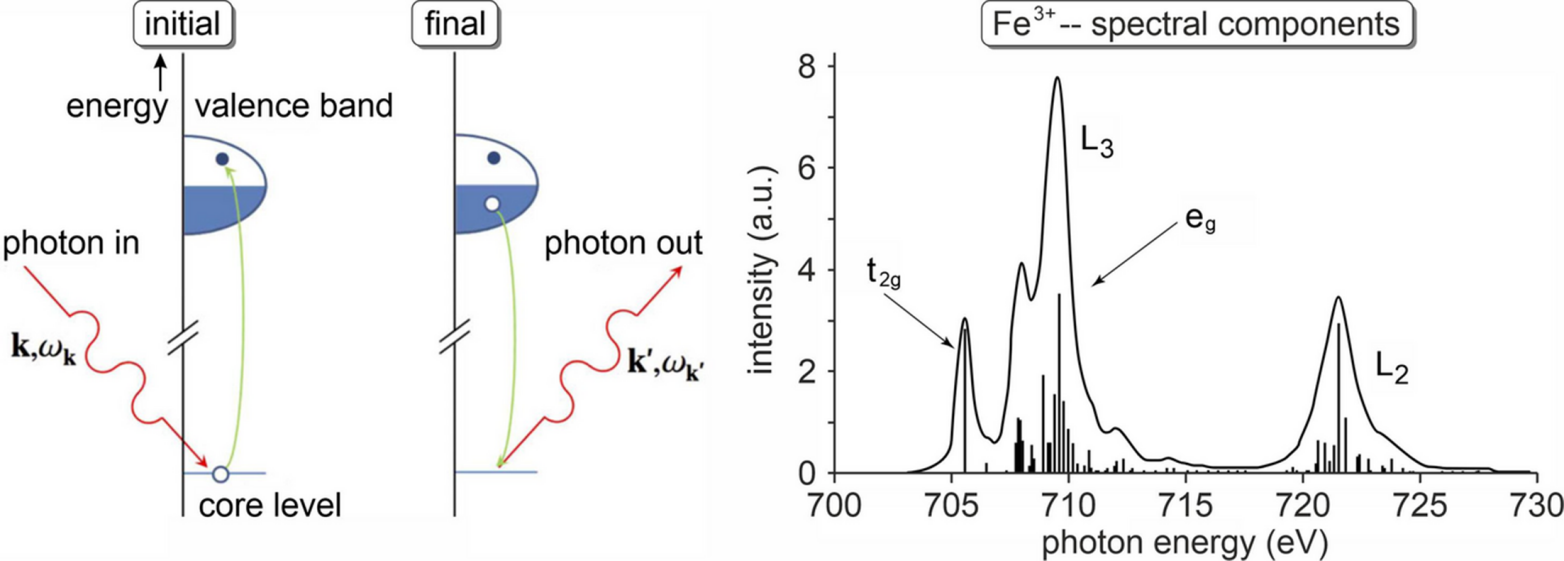
Schematic of the direct RIXS process (left) and a typical fluorescence spectrum at the *L*_2,3_-edge of a ferric system (right). Multiple, closely spaced transitions (‘needle’ peaks) are convolved with the instrumental response in the *L*_3_ range (703–713) eV and the weaker *L*_2_ band (717–727) eV.

Soft X-ray spectra, obtained by diffraction from grating-like optical elements, unveil the electronic structure especially of molecules (Wernet, 2019 ▸) in terms of charge and spin. Element-specific information is obtained from peak positions and their shifts due to characteristic binding energies in a given chemical environment. An excellent energy resolution of the wavelength-dispersive instrument, at a sufficiently high photon flux, is required to deconvolve lines separated on the meV scale in ‘photon hungry’ resonant inelastic X-ray scattering (RIXS) (Revelli *et al.*, 2019 ▸; Gilmore *et al.*, 2021 ▸; Rahn *et al.*, 2022 ▸; Higley *et al.*, 2022 ▸; Söderström *et al.*, 2024 ▸; Mitrano *et al.*, 2024 ▸; de Groot *et al.*, 2024 ▸). Time-resolved experiments on the pico- or even femto-second scale benefit from an efficient detection as well (Lu *et al.*, 2020 ▸; Jost *et al.*, 2025 ▸; Johnson & Staub, 2025 ▸). 2*p* → 3*d* excitation and subsequent dipole-allowed and hence relatively intense emission at the *L*-edge of transition metals is of particular interest (Guo *et al.*, 2024 ▸). Fig. 1 ▸ shows the physical principle of direct RIXS (Pavarini *et al.*, 2016 ▸) and an example spectrum for Fe (Wasinger *et al.*, 2003 ▸).

At present, spectrometers at synchrotron or free-electron laser facilities but also in laboratories make use of optimized variable-line-space (VLS) gratings (Urpelainen *et al.*, 2017 ▸; Pietzsch *et al.*, 2018 ▸; Meier *et al.*, 2025 ▸; Yamamoto *et al.*, 2025 ▸; Sun *et al.*, 2025 ▸; Schlappa *et al.*, 2025 ▸). However, phase distortions, caused by figure errors of the substrate, often limit the resolving power in practice. Corrective methods comprise use of refractive phase plates (Seiboth *et al.*, 2017 ▸) and ion beam figuring (Ice *et al.*, 2000 ▸; Shurvinton *et al.*, 2024 ▸). In this paper, we adapt the two-dimensional (2-D) groove structure of an off-axis soft X-ray reflection zone plate (RZP) (Mitzner *et al.*, 2013 ▸; Kroll *et al.*, 2016 ▸) to compensate surface irregularities (Probst *et al.*, 2020 ▸; Kubec *et al.*, 2022 ▸) by a diffractive wavefront corrector (DWC). In Section 2, we describe how the profile of the substrate can be modeled, and sketch the layout of the instrument in Section 3. The algorithm to compute the DWC is detailed in Section 4, followed by performance simulations in Section 5 and the error budget in Section 6. We conclude in Section 7.

## 2. Grating substrate characterization

**Figure 2:**
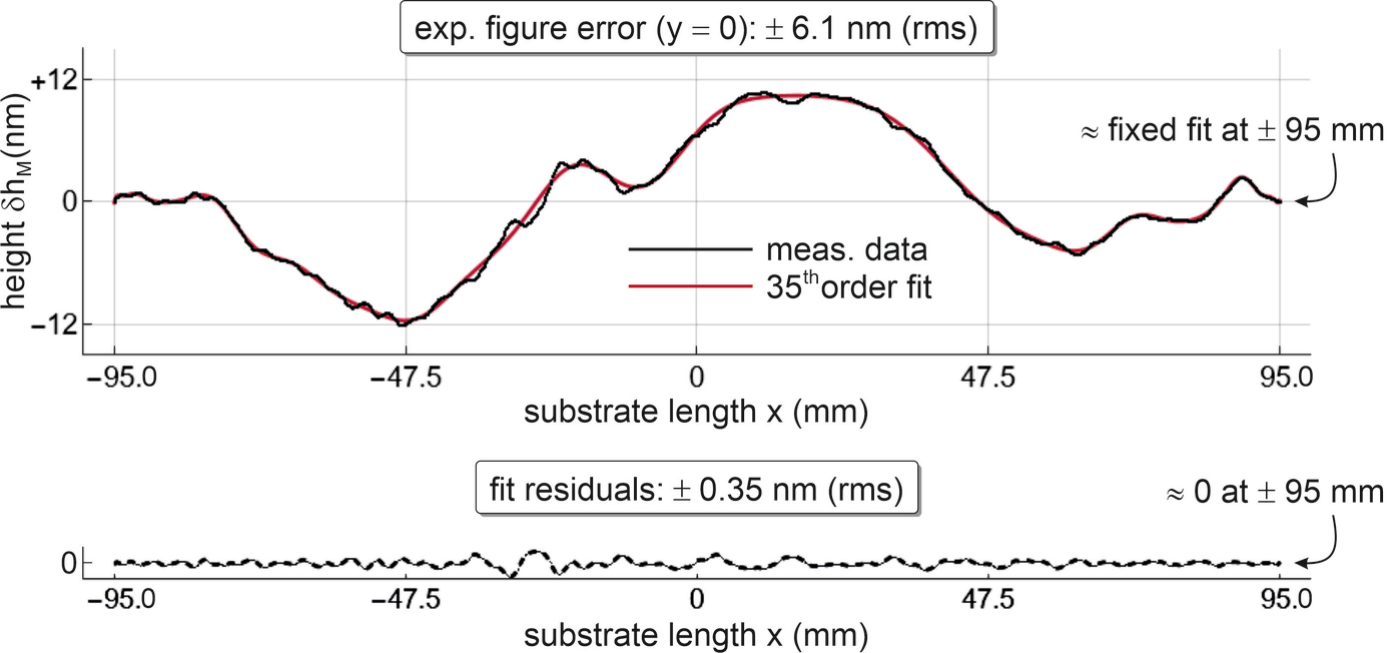
Experimental on-axis height deviation δ*h*_*M*_(*x*, 0) from the spherical form of the grating substrate in the longitudinal direction at a sampling rate of 5 mm^−1^ (black) and its polynomial fit in the Legendre basis of 35th order (red). Residuals describe the difference between model and data.

In the first instance, the super-polished, spherical Si substrate of 220 mm × 50 mm is probed *ex situ* using the nanometre optical component measuring machine (NOM) at the Helmholtz-Zentrum Berlin (Siewert *et al.*, 2012 ▸). This highly precise, calibrated (Siewert *et al.*, 2010 ▸) device probes the local slope υ at a measurement uncertainty of ±10^−2^ arcsec (r.m.s.) along the central surface line at ∼10^3^ positions −95 mm ≤ *x* ≤ +95 mm (length *L*) in steps of 0.2 mm by a reflected laser beam, detected in an autocollimator unit. By discrete integration of the data υ(*x*), the height profile can be obtained, and subtraction of a fitted sphere yields the radius of curvature, as it will be specified below in Section 3. For the tangential slope deviation of ±0.11 arcsec (r.m.s.) on average, the on-axis (*y* = 0) figure error δ*h*_*M*_ varies within ±11.4 nm (peak-to-valley, P-V) or ±6.11 nm (r.m.s.) along the substrate in Fig. 2 ▸.

The sampling period above records the full range of low spatial frequencies (LSF) up to 2.5 × 10^−3^ µm^−1^, according to the Nyquist–Shannon theorem^1^, and pointwise interpolation is possible, in principle. However, to apply the fast, semi-analytic algorithms for wavefront correction (Section 4) and subsequent ray tracing (Section 5), the experimental data for δ*h*_*M*_(*x*, 0) should be approximated by an appropriate, differentiable fit function. We choose a linear, high-order combination of orthonormal Legendre polynomials *P*_*n*_(*x*), which are defined via Rodrigues’ formula on the interval 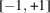,
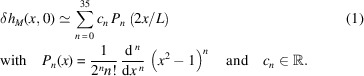
This model suppresses oscillatory artifacts at the boundaries (±95 mm) but filters the real surface to lower spatial frequencies, as depicted in Fig. 2 ▸. Compared with the magnitude of δ*h*_*M*_, the fit residuals are small, with an amplitude of a few angstrom (r.m.s.). For their standard deviation of ±0.35 nm (Fig. 2 ▸) and the mean grazing angle of 4° (Section 3), the statistical wavefront error of ±λ_0_/36 at the wavelength λ_0_ corresponds to a Strehl ratio of 97%. The surface appears sufficiently smooth to such X-rays after subtraction of the fit, which is accomplished by adapting the grating lines (Section 4), and nearly diffraction-limited resolution can be achieved at the design energy (Section 5).

**Figure 3:**
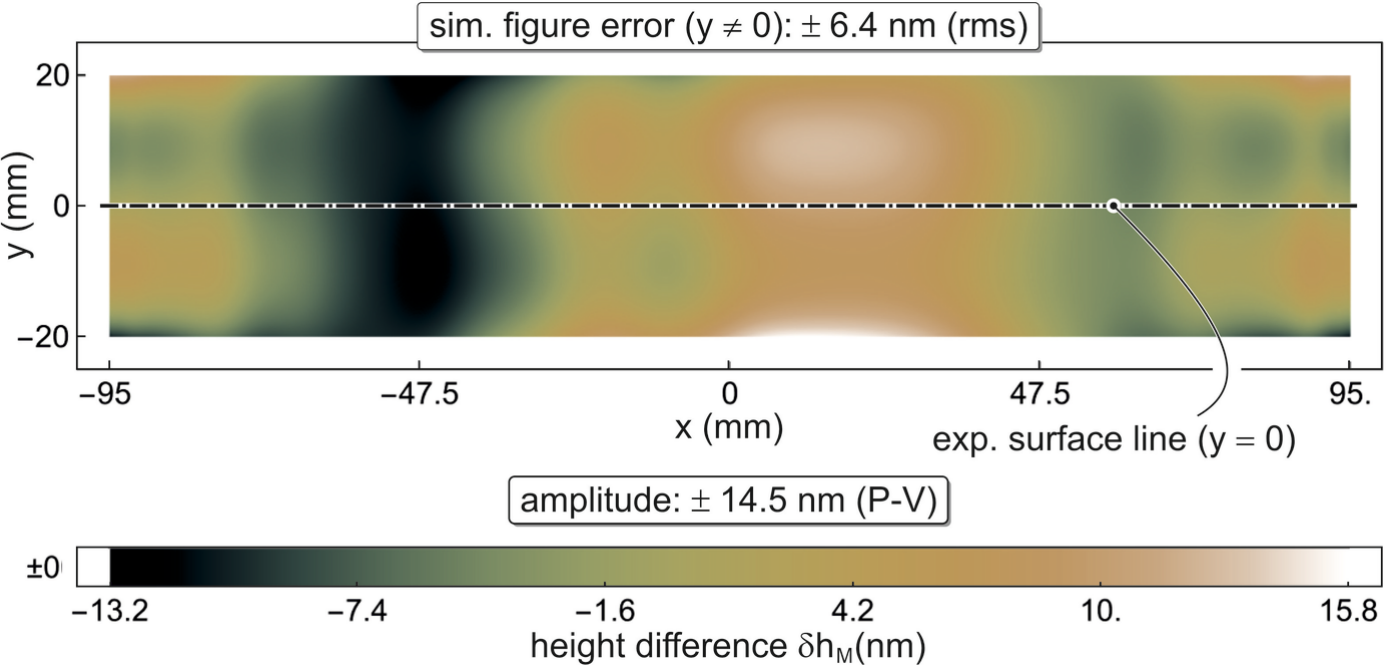
Simulated height deviation δ*h*_*M*_(*x*, *y*) from the spherical form of the grating substrate at off-axis positions (*y* ≠ 0), as an extension of the experimental on-axis (*y* = 0) surface line (Fig. 2 ▸). The typical amplitude of ±6.44 nm (r.m.s.) corresponds to a peak-to-valley (P-V) range of ±14.5 nm.

Real mirrors are characterized by a 2-D figure error δ*h*_*M*_(*x*, *y*) which, in general, differs at *y* ≠ 0 from the axial surface line δ*h*_*M*_(*x*, 0). The full height information not only in longitudinal but also transverse direction must be taken into account for proper wavefront correction. The extension to arbitrary 2-D profiles and thus asymmetric DWC structures is straightforward and should be realized in practice by measuring multiple off-axis surface lines (Probst *et al.*, 2020 ▸). In the absence of such data presently, we augment the on-axis profile with a simulated waviness in terms of 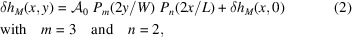
which is defined for |*y*| ≤ *W*/2 and coincides at *y* = 0 with the measured profile from Fig. 2 ▸ for the odd transverse coefficient *m*. Though typical for figure errors at low spatial frequencies, the off-axis model from equation (2) – or a linear combination of low-order polynomials^2^ – is by far not representative for arbitrary 2-D surface perturbations. The theory of diffractive wavefront correction, as outlined in the following, is therefore limited to this tested class of low-order off-axis figure error models. Since the amplitude 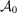 = 11.4 nm equals the halved on-axis P-V above, δ*h*_*M*_(*x*, *y*) varies on a similar level, typically within ±6.4 nm (r.m.s.), as visualized in Fig. 3 ▸.

## 3. Spectrometer layout

**Figure 4:**
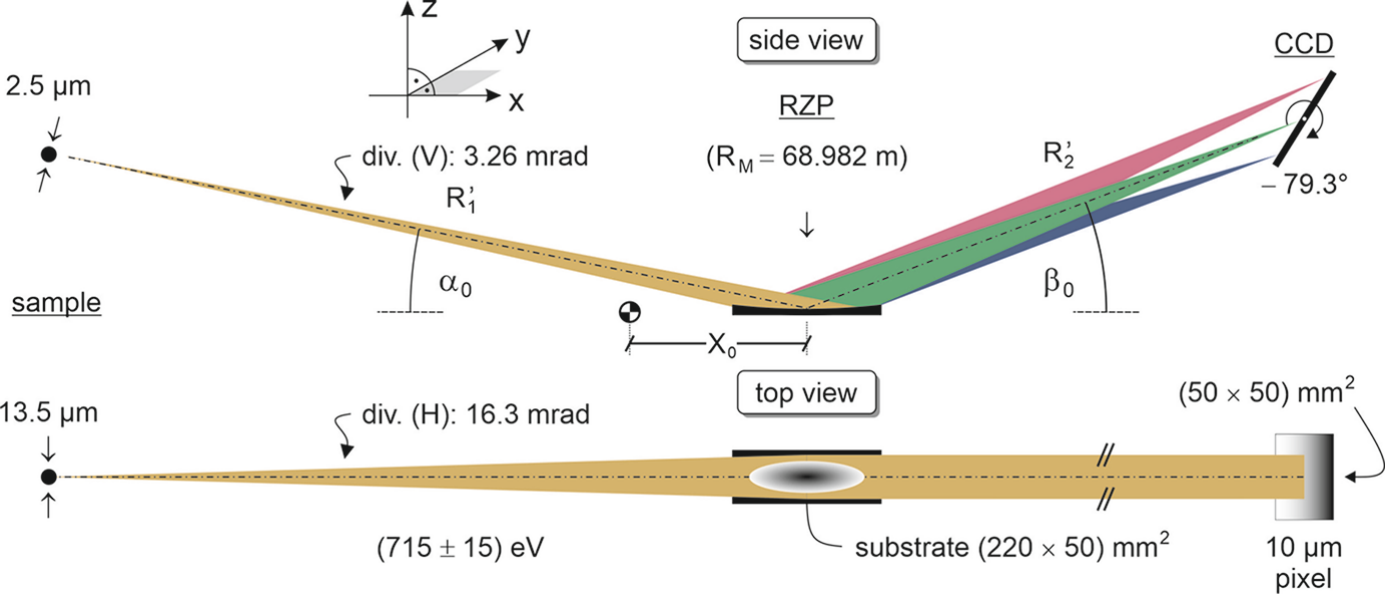
Schematic of the spectrometer. The divergent emission from the point-like source (sample) is captured by the RZP, which combines ‘point to collimated line’ focusing and dispersion on the CCD (or CMOS) camera. The design parameters are partially adopted from Pietzsch *et al.* (2018 ▸).

An off-axis RZP (Mitzner *et al.*, 2013 ▸; Kroll *et al.*, 2016 ▸; Probst *et al.*, 2020 ▸) is commonly characterized by the geometry, in terms of the entrance (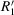) and exit (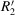) arm with associated grazing angles (α_0_, β_0_), as sketched in Fig. 4 ▸.

The nominal energy *E*_0_ and the displacement *X*_0_ of the diffractive aperture (length *L*, mean width *W*) from the optical center of the RZP define the central density of grating lines *d*_*l*_(0) on the substrate of radius *R*_*M*_ from Section 2. The design is summarized in Table 1 ▸.

For an incoherent, Gaussian-shaped source of 13.5 µm × 2.5 µm (H × V) in size (FWHM) and the magnification *M* = 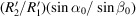, we expect at *E*_0_ in the absence of aberrations a focal line width of 2.4 µm in the dispersive direction (V), including diffraction at the RZP aperture. To avoid detector-limited resolution, the surface normal of the CCD (or CMOS) camera with an active area of 50 mm × 50 mm at a pixel size of 10 µm is therefore inclined by −79.3° with respect to the optical axis (Pietzsch *et al.*, 2018 ▸), also matching the chromatic aberration approximately, as depicted in Fig. 4 ▸. This tilted mount will allow for a moderate, at least 1.5-fold, oversampling of spectral lines around the design energy, and the full Fe *L*_2,3_ energy range of (700–730) eV can be recorded due to an – almost constant – dispersion ∂*E*/∂*z*_det_ = −0.67 eV mm^−1^, where *z*_det_ denotes the local *z*-axis on the detector.

To obtain the profile function *z*_*M*_(*x*, *y*) of the substrate, we add the surface imperfections δ*h*_*M*_(*x*, *y*), as specified by equation (2) and shown in Fig. 3 ▸, as a small distortion to the ideal spherical form, 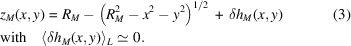
As the zero average of δ*h*_*M*_(*x*, *y*) in equation (3) implies, the focal lines will be centered at their nominal position for an ideal sphere. The optical path length 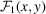 of a photon (*E*_0_) propagating from the source to an arbitrary position (*x*, *y*) on the RZP is given by (Probst *et al.*, 2020 ▸) 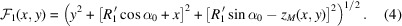
The distance 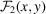 from the RZP – or DWC, as the generalized RZP on an irregular substrate – to the corresponding point on the focal line (*y* = constant) is calculated similarly, 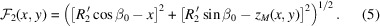
By this definition, the RZP collimates the divergent source emission in one dimension (1-D) to a straight (at *E*_0_) focal line parallel to the *y*-axis (Fig. 4 ▸). Relative to the axial ray along *y* = 0 (dash-dotted line in Fig. 4 ▸), the path difference 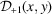 in the (+1)st diffraction order can be written as 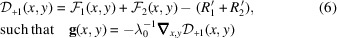
with λ_0_ = *hc*/*E*_0_ (Planck constant *h*, vacuum velocity of light *c*) yields the vector field **g**(*x*, *y*) of the 2-D grating line density (Probst *et al.*, 2020 ▸). Whereas 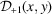 directly leads to the binary groove structure of the phase-corrected RZP in Section 4, its gradient **g**(*x*, *y*) will be used in Section 5 to simulate the performance.

## 4. Wavefront correction

Several techniques have been proposed and realized to compensate for the irregular figure error of the optical surface. Properly shaped, refractive phase plates work well in transmission at hard X-rays of several keV, due to moderate or even negligible absorption (Seiboth *et al.*, 2017 ▸). Mirrors can be evened out by ion beams, depositing thin layers of atoms into the troughs or sags of the reflective element (Ice *et al.*, 2000 ▸; Shurvinton *et al.*, 2024 ▸). Aside from these additive and potentially time-consuming methods, an adapted line density distribution makes sense especially for diffractive components, as it was demonstrated recently for a 1-D VLS grating (Kubec *et al.*, 2022 ▸). In the following, we correct the spherical substrate from Section 2 by an inherently 2-D, holographic approach (Probst *et al.*, 2020 ▸). For 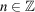, the Fresnel zones 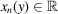 are solutions of the implicit, nonlinear relation 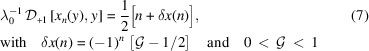
as the groove-to-period ratio of the binary diffractive structure. The roots to equation (7) with the continuously differentiable function 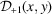 can be found by an appropriate algorithm based on, for example, the Newton method, as implemented in computer algebra systems such as *Mathematica*^TM^: using on-axis solutions *x*_*n*_(0) as starting points, the Fresnel condition (7) is evaluated for a discrete set of ∼10^2^ equally distributed sampling points *y*_*i*_ with 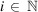 across the local width of the RZP aperture (∼40 mm). Combining the data {*x*_*n*_(*y*_*i*_), *y*_*i*_} of two subsequent Fresnel zones then approximates each grating groove by a polygon – a format suitable for lithography systems. The trapezoidal shape of the RZP and the peculiar form of the Fresnel zones at the lower edge (*x* ≳ −95 mm) as well as in the upper left and right corners (*x* ≲ +95 mm) lengthen the code and require manual settings for boundary zone numbers. In its present form, the computation of the full DWC structure with 4.1 × 10^5^ grooves takes several hours on a 4 GHz CPU (single core), assuring numerical precision at the sub-nm level, which can be regarded in relation to the physical and technical constraints:

(i) As an uppermost limit, the Maréchal criterion (λ_0_/14), applied to the average line density *d*_*l*_(0) from Table 1 ▸, would accept an accidental, statistical tolerance of about ±20 nm (r.m.s.) for the longitudinal position of the grooves, *i.e.* along the *x*-coordinate.

(ii) A – preferably much – better precision is desired nonetheless, and, in fact, the practical accuracy is given by the placement error of modern electron beam (∼1 nm) or laser (∼2 nm) lithography at least in the *x*-direction, across the full grating width −*W*/2 ≤ *y* ≤ +*W*/2.

**Figure 5:**
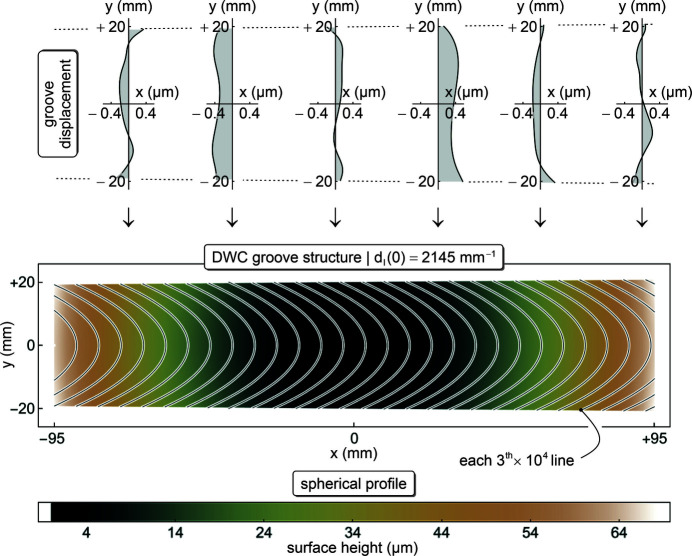
Groove structure (white lines) of the DWC on a spherical substrate (color code) in top view (bottom). The displacement from the grooves of a regular RZP is displayed for selected lines.

The grating lines of the DWC are displaced from those of the regular RZP (δ*h*_*M*_ → 0) on a scale of ∼10^−1^ µm, as shown in Fig. 5 ▸. The mean difference (≃0) between the Fresnel zones *x*_*n*_(*y*) of the DWC and the uncorrected RZP and its magnitude of ±0.25 µm (r.m.s.) reflect the zero leveling and average amplitude of the figure error 〈δ*h*_*M*_(*x*, *y*)〉_*L*_, respectively.

The latter has been measured in terms of the tangential slope υ(*x*), fitted along the 1-D surface line (*y* = 0), and simulated by means of an additive, asymmetric function for *y* ≠ 0. Hence, the residual roughness (Fig. 2 ▸) of ±0.35 nm (r.m.s.), though present for −*W*/2 ≤ *y* ≤ +*W*/2, takes effect only along *x* within this model, leading to minor scattering in the meridional direction as estimated by the Strehl ratio in Section 2. The loss of photons and potential blur of the focal line in the sagittal (*y*) direction, caused by the 2-D nature of the roughness on a real surface, can be neglected even more due to the ‘forgiveness factor’ 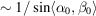 under grazing incidence and diffraction (Cash, 1987 ▸; Urpelainen *et al.*, 2017 ▸), suppressing the transverse deflection of X-rays to only ∼7% of the tangential scattering for the mean 〈…〉 of design angles (α_0_, β_0_) from Table 1 ▸.

The parameters of the laminar grating profile are optimized using rigorous coupled wave analysis (RCWA), assuming an Au coating with a reflectivity around 27.7%. For an etch depth of 6 nm and a groove-to-period ratio of 61%, we find an almost constant (+1)st order efficiency 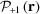 = (6.4 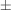 0.1)% across the RZP field (Fig. 5 ▸) – even at the edges (*y* ≃ ±20 mm), where the grating lines of high density (up to 5.3 × 10^3^ mm^−1^) are illuminated under inclined incidence, *i.e.* the RZP benefits from low-loss conical diffraction. The variation of 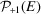 with the photon energy *E* by ±0.04% can be neglected within the 30 eV band as considered in this work. Of particular advantage for RIXS applications is the weak polarization dependence: we expect a cosine-like modulation amplitude of ±0.05% (P-V), at a higher efficiency for transverse electric waves compared with the transverse magnetic mode. Other diffraction orders, including the 0th with 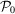 = 29.3%, are separated from the camera by the off-axis segment (*X*_0_) of the zone plate.

## 5. Performance simulation

**Figure 6:**
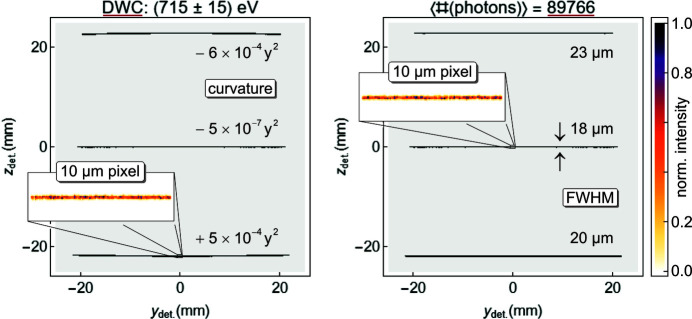
Detector view of a simulated spectrum, using the DWC. The weak focal line curvature at off-design energies (left) is corrected, and all photons in the (+1)st order are captured by the camera (right). The FWHM is given in µm. Cut-outs show zoom views on the 10 µm pixel scale.

A customized ray-tracing code is implemented in the *Mathematica*^TM^ add-on package *Optica*^TM^. The 2-D grating line density **g**(*x*, *y*) of the DWC follows from equation (6) with 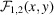

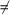
 0 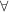 (*x*, *y*), 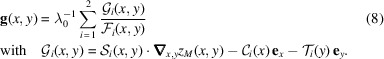
In equation (8), the auxiliary functions 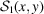, 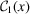 and 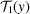 for an incident ray are given as 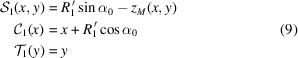
whereas the terms 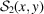, 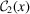 and 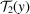 describe the exit path from the DWC to the CCD, 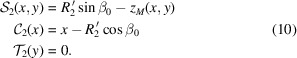
Since *z*_*M*_(*x*, *y*) was implemented as an analytical, continuously differentiable expression in Section 2, the optical scheme can be simulated precisely and fast in the *Optica*^TM^ software environment. Fig. 6 ▸ displays the result for an input spectral distribution of three ‘delta peaks’ at (715 ± 15) eV.

In agreement with the path function 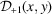 in equation (6), a straight [the residual curvature in *z*_det_(*y*) of a few 10^−7^*y*^2^ lies within statistical error margins and can be neglected], collimated focal line is obtained for *E*_0_. Diffraction at the grating aperture contributes 3 µm to the 1-D focal line width of 17.8 µm, just 10% above the minimum for ideal optics, indicating a well compensated figure error at the design energy. The confined, symmetric Gaussian line shape is almost maintained (*i.e.* without coma tails) for photon energies *E* ≠ *E*_0_, just slightly broadened to at most (20–23) µm at the outer edges of the range (700–730) eV, and slightly ‘bent’ to no more than about *z*_det_(*y*) ≃ *z*_0_(*E*) ± 5 × 10^−4^*y*^2^ at *E*_0_ ± 15 eV, where *z*_0_(*E*) denotes the energy-dependent line position at *y* = 0. This distortion can be easily eliminated during post-processing of the raw CCD data, *i.e.* by subtraction of an appropriate fit. Finally, the flattened spectral lines can be read out along the pixel rows in horizontal (H) direction (Fig. 6 ▸). We analyze resolving power and photon flux:

(i) With the nominal settings for source and pixel size in Section 3, the simulation of the DWC from Fig. 5 ▸ reveals a resolving power *E*/Δ*E* ≃ 6.0 × 10^4^ at *E*_0_, which degrades to (5.0 ± 0.3) × 10^4^ toward the edges of the *L*_2, 3_ range at *E*_0_ ± 15 eV. To illustrate the effect of wavefront correction within that energy window under otherwise identical conditions, we simulate a regular RZP assuming δ*h*_*M*_(*x*, *y*) → 0 in equation (6) on the real, wavy substrate: the focal line at *E*_0_ would be blurred across ∼7 pixels and the mean resolving power is reduced to (2.3 ± 0.1) × 10^4^. In any case, however, the full flux from the (+1)st diffraction order can be recorded within the camera aperture, due to the sagittal collimation (Fig. 4 ▸) by the 2-D curved grating lines.

(ii) In contrast, a wavefront-corrected 1-D VLS grating (Kubec *et al.*, 2022 ▸) of the same on-axis line density *d*_*l*_(*x*) = |*g*_*x*_(*x*, 0)| with *g*_*y*_(*x*, *y*) ≡ 0 in equation (6) would induce widespread, curved focal lines: in fact, the diffracted beam propagates almost unfocused in the sagittal direction, and only 41% of the photons in the (+1)st order will be captured by the camera. Besides, a strong concave slope is introduced at all photon energies, evaluated to *z*_det_(*y*) ≃ *z*_0_(*E*) − 2 × 10^−3^*y*^2^. After straightening (image processing, see above), the averaged resolving power between 700 eV and 730 eV could be nonetheless approximately maintained to (4.9 ± 0.1) × 10^4^ – only 8% less than for the 2-D DWC from Fig. 5 ▸. If, in addition, the information about the substrate’s figure error is also omitted (Pietzsch *et al.*, 2018 ▸) via δ*h*_*M*_(*x*, *y*) = 0 in the expression for *z*_*M*_(*x*, *y*) as defined in Section 2, the focal lines are blurred to (46 ± 1) µm and the resolving power further degrades to *E*/Δ*E* = 2.3 × 10^4^, virtually constant within the Fe *L*_2, 3_-edge energy window.

(iii) We define a figure of merit, 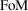

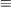

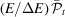
, where the overall transmission 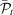 of the instrument multiplies the (+1)st order diffraction efficiency 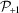 from Section 4 with the geometric capture on the detector^3^. The mean resolving power of the corrected (superscript ‘c’) 2-D RZP (*i.e.* the DWC in Fig. 5 ▸) is 2.29 times higher than that of the regular (superscript ‘r’) 1-D VLS grating (Pietzsch *et al.*, 2018 ▸). Combined with a gain of 2.46 in 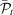, *i.e.* the photon flux enabled by collimation (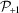 is assumed to be equal), we find a performance enhancement 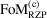 = 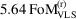 for the source (V) and pixel size as specified in Section 3.

**Figure 7:**
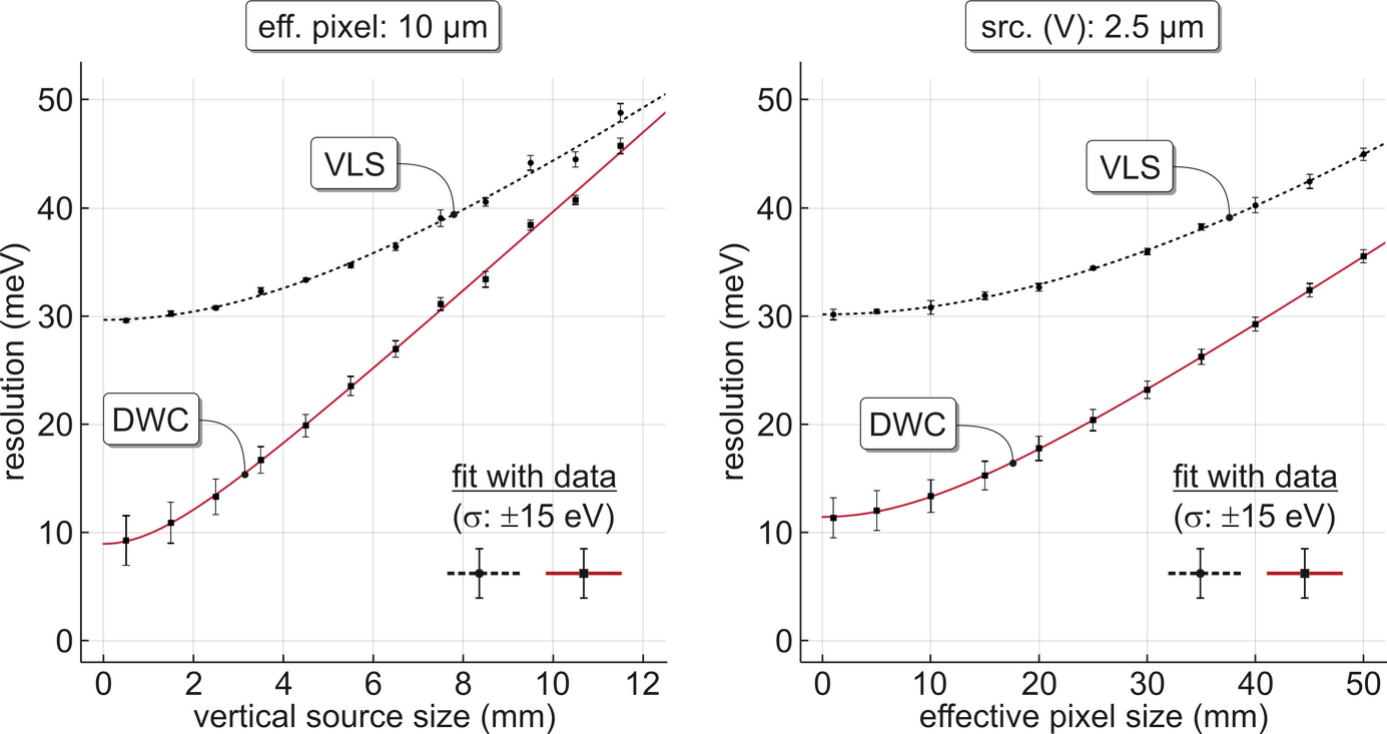
Energy resolution as a function of the source size (V) for fixed effective pixels at 10 µm (left) and in dependence on the effective pixel size for the nominal source diameter (right). Simulated data for the uncorrected VLS grating and the DWC are fitted by the same model (dashed black and red solid lines), as described in the text. Error bars refer to the variation (σ) within (715 ± 15) eV.

Source and pixel size can affect the resolution notably. In Fig. 7 ▸, one of them is varied while the other one is fixed.

The simulated data are fitted by optimizing the coefficients *c*_*i*_ in the model 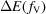 = 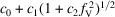, where *f*_V_ represents the feature (*i.e.* source or pixel) size in the dispersive (V) direction.^4^ More than the regular 1-D VLS grating, the DWC benefits from a small source (V), at an FWHM close to the nominal value of 2.5 µm (Section 3), and from small pixels of ∼10 µm.

## 6. Error budget

Under real operating conditions, the intrinsic limitations of technical devices used for alignment, but also external influence from a thermal drift of components or their mechanical creep, affect orientation and position of components. We estimate the tolerance of the overall instrument in terms of the resolving power, regarding potential misalignments of source, DWC and detector by means of Monte Carlo ray tracing including pixel size and diffraction limit, whereas the grating efficiency is analyzed for typical fabrication errors using RCWA.

If the entrance arm length is slightly modified by 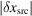

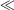

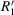
 (Table 1 ▸), the focal line width at *E*_0_ expands as 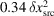 relative to its minimum (Section 5). Hence, to preserve 98 (95)% of the full resolving power *E*/Δ*E*, the source should be placed within ±0.25 (0.40) mm around the nominal distance 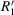. For large shifts beyond ±1 mm, the resolution degrades in proportion to δ*x*_src_.

As holographic optical elements, RZPs must be mounted precisely according to their design parameters (Table 1 ▸). However, residual misalignment will degrade in particular the resolving power of the DWC, which is just a generalized RZP on a wavy substrate. Without loss of generality, we perform Monte Carlo simulations (ray tracing) at *E*_0_ for 3-D tilts around the geometrical center **r** = 0 of the DWC and translations along its local axes (Fig. 5 ▸). The focal line width blurs approximately with the second order of small adjustment errors, corresponding to a few percent loss in *E*/Δ*E*, in each of the angular (δ**φ**) or spatial (δ**r**) dimensions, and expands in the linear regime for large deviations from the designed values. In Table 2 ▸, we summarize this error budget for two tolerance levels on the resolving power relative to the maximum (Section 5). The accuracy required for ‘pitch’ δφ_*y*_ and ‘yaw’ δφ_*z*_ should especially be addressed by state-of-the-art six-axis nanopositioning. The margins and the corresponding levels in Table 2 ▸ hold for each degree of freedom individually, *i.e.* if all others are perfectly aligned. In practice, however, multiple perturbations can occur simultaneously. Since these errors accumulate in general, care must be taken. Avoiding further loss in the observed resolving power *E*/Δ*E* might thus require even tighter margins in the relevant dimensions.

Among all possible displacements of the camera, only the rotation ϕ_*y*_ around the *y*-axis and the translation δ*x*_det_ along the local optical axis are relevant. The inclination angle of the detector affects the sharpness of the focal lines at off-design energies, and we simulate the mean resolving power within *E*_0_ ± 15 eV for variations δϕ_*y*_ of at most ±1° around the optimum 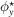 = −79.3° from Section 3. Taking into account the finite pixel size, small misalignments δϕ_*y*_ up to ±0.1° nearly (∼99%) maintain the maximum in *E*/Δ*E*, whereas, for large deviations beyond ±0.3°, *E*/Δ*E* decreases linearly with δϕ_*y*_. The large exit arm length 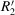 from Table 1 ▸ implies a relaxed positioning of the detector: convolved with the pixel size again, the resolving power at *E*_0_ degrades as 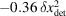 relative to its maximum for small moves |δ*x*_det_| ≲ 0.5 mm around the nominal distance 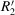.

The groove depth *t*_g_ and ratio *s*_g_ of the DWC vary in the vicinity of their optimal values (index 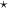) from Section 4 as a square law, too. Around the maximum 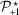 = 6.4%, the simulation yields 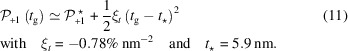
98 (95)% of 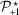 are maintained within the tolerance δ*t*_g_ of ±0.6 (0.9) nm. The less critical dependence on the relative groove width 0 ≤ *s*_g_ ≤ 1 of the laminar profile is modeled in analogy, 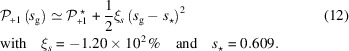
98 (95)% of 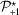 are diffracted within a range δ*s*_g_ of ±0.046 (0.073). Other imperfections such as trapezoidal shapes, surface contamination or (line edge) roughness are not considered here. Stitching errors, sometimes introduced during electron beam or laser lithography, might disturb the phase coherence of the wavefront. However, the RZPs manufactured over the years at HZB and at companies like NOB Nanooptics Berlin, for instance, are practically free from such structural discontinuities. According to our experience, there is no observable impairment of performance.

We further note that the heat load problem, leading to thermal expansion of the DWC substrate under intense irradiation, is not relevant for reflective (and therefore weakly absorbing) X-ray optics which can be even cooled efficiently. Gravitational or other bending and torsion can be ruled out by a sufficiently thick, monocrystalline Si substrate (≳10 mm), fixed at multiple points.

## 7. Conclusion

An advanced soft X-ray spectrometer for beamlines at synchrotron or free-electron laser facilities, utilizing an aberration-reduced, 2-D RZP with modified grating grooves on a spherical substrate with figure errors, is proposed and simulated in terms of its performance. The mean resolving power *E*/Δ*E* = (5.3 ± 0.6) × 10^4^ in the energy range 700 eV ≤ *E* ≤ 730 eV is enhanced by a factor of 2.3 while the photon flux on the detector is almost 2.5 times higher, compared with an analog 1-D VLS grating (Pietzsch *et al.*, 2018 ▸) without wavefront correction and collimation.

We define the phase function and derive the vector field for the DWC on a curved and wavy substrate with measured (on-axis) and simulated (off-axis) height profile perturbations. Based on this information, we sketch the formalism for computing the groove structure, which can be regarded as a computer-generated hologram on an irregular 3-D surface (Probst *et al.*, 2020 ▸): at a central line density around 2.15 × 10^3^ mm^−1^, the position and shape of the grooves differ from those of a regular RZP or VLS on the scale of a half to one period, *i.e.* up to a few 100 nm. The required resolution and precision will pose the question of appropriate fabrication – using either electron-beam lithography as the present state of the art or fast and low-cost two-photon polymerization (‘direct laser writing’) in the future. With a total optical path length of 7.55 m (Pietzsch *et al.*, 2018 ▸) and a fixed-focus constant (*c*_ff_) of 2.28, a dispersion between −0.64 eV mm^−1^ and −0.70 eV mm^−1^ is achieved in the flat but tilted focal plane. We simulate the performance for an incoherent source of 13.5 µm × 2.5 µm (H × V) and use a strongly inclined detector (−79.3°) with a pixel size of 10 µm, moderately oversampling the intensity distribution (FWHM) of the focal lines by a factor of ≲2. The instrumental error budget for fabrication and positioning tolerances indicates feasible but tight margins, especially on the angular accuracy (∼µrad) with which the DWC should be aligned.

Applications include, amongst others, soft X-ray emission (XES) and absorption (XAS) spectroscopy, either static or time-resolved (Jost *et al.*, 2025 ▸) at, for example, free-electron lasers (Lu *et al.*, 2020 ▸; Johnson & Staub, 2025 ▸). Polarization is preserved under grazing incidence, which makes the customized DWC conception also suitable for resonant elastic (REXS) and inelastic (RIXS) X-ray scattering (Higley *et al.*, 2022 ▸), probing even phonon excitations in solids on a scale of ∼10^−2^ eV (Pavarini *et al.*, 2016 ▸). Future efforts might access the *L*-edge of other transition metals, in particular of _29_Cu and _30_Zn with an energy of ∼1 keV, to measure the dispersion relation over an extended range of momenta (Pavarini *et al.*, 2016 ▸). Due to a large acceptance solid angle, in our version 5.3 × 10^−5^ sr, and high diffraction efficiency of 6.4% – which might be further enhanced using blazed profiles (Hofhuis *et al.*, 2025 ▸; Fernández Herrero *et al.*, 2025 ▸) – the *K*-edge of light elements from _3_Li to _5_B, or from _6_C to _8_O (Söderström *et al.*, 2024 ▸; Guo *et al.*, 2024 ▸) can be also studied by phase-corrected, wavelength dispersive soft X-ray spectroscopy at ∼ meV resolution.

## Figures and Tables

**Table 1:** RZP design energy *E*_0_, entrance (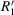) and exit (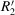) arm lengths with angles (α_0_, β_0_), and the off-axis shift *X*_0_ with a line density *d*_*l*_(0). *R*_*M*_ denotes the radius of the grating (size *L* × *W*)

*E*_0_	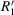	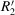	α_0_	β_0_	*X*_0_	*d*_*l*_(0)	*R*_*M*_	*L* × *W*
715 eV	2.449 m	5.100 m	2.405°	5.498°	1.139 m	2145 mm^−1^	68.982 m	190 mm × 40 mm

**Table 2:** The DWC error budget for angular (δ**φ**) and translational (δ**r**) dimensions of misalignment

Level	δφ_*x*_	δ*r*_*x*_	δφ_*y*_	δ*r*_*y*_	δφ_*z*_	δ*r*_*z*_
98%	±241 µrad	±350 µm	±4.61 µrad	±2.25 µm	±0.81 µrad	±6.11 µm
95%	±412 µrad	±555 µm	±7.32 µrad	±3.57 µm	±1.29 µrad	±9.70 µm

## Data Availability

Data and code underlying the results may be obtained from the authors upon request.

## Footnotes

1 Higher spatial frequencies, including negligible micro-roughness (on the order of a few angstrom), do not contribute to geometrical aberrations but rather reduce the peak intensity while the scattering background is increased.

2 Other sufficiently small coefficients (*m*, *n*) ≲ 5 have no significant effect on the results (Section 5), *i.e.* the DWC still works for such deformations. This does not imply necessarily a general validity for any real functions δ*h*_*M*_(*x*, *y*).

3 The quantum efficiency of the detector, filters, or other technical details are not included in the transmission .

4 For the simulation on the left in Fig. 7 ▸, the horizontal (H) source width is adjusted in proportion. On the right, the squared pixels are specified by their edge length. Evaluations are performed with ≲10^4^ rays per photon energy.
